# Assessing the Usability of a Clinical Decision Support System: Heuristic Evaluation

**DOI:** 10.2196/31758

**Published:** 2022-05-10

**Authors:** Hwayoung Cho, Gail Keenan, Olatunde O Madandola, Fabiana Cristina Dos Santos, Tamara G R Macieira, Ragnhildur I Bjarnadottir, Karen J B Priola, Karen Dunn Lopez

**Affiliations:** 1 College of Nursing University of Florida Gainesville, FL United States; 2 College of Nursing University of Iowa Iowa City, IA United States

**Keywords:** usability, heuristic, clinical decision support, electronic health record, expert review, evaluation, user interface, human-computer interaction

## Abstract

**Background:**

Poor usability is a primary cause of unintended consequences related to the use of electronic health record (EHR) systems, which negatively impacts patient safety. Due to the cost and time needed to carry out iterative evaluations, many EHR components, such as clinical decision support systems (CDSSs), have not undergone rigorous usability testing prior to their deployment in clinical practice. Usability testing in the predeployment phase is crucial to eliminating usability issues and preventing costly fixes that will be needed if these issues are found after the system’s implementation.

**Objective:**

This study presents an example application of a systematic evaluation method that uses clinician experts with human-computer interaction (HCI) expertise to evaluate the usability of an electronic clinical decision support (CDS) intervention prior to its deployment in a randomized controlled trial.

**Methods:**

We invited 6 HCI experts to participate in a heuristic evaluation of our CDS intervention. Each expert was asked to independently explore the intervention at least twice. After completing the assigned tasks using patient scenarios, each expert completed a *heuristic evaluation checklist* developed by Bright et al based on Nielsen’s 10 heuristics. The experts also rated the overall severity of each identified heuristic violation on a scale of 0 to 4, where 0 indicates no problems and 4 indicates a usability catastrophe. Data from the experts’ coded comments were synthesized, and the severity of each identified usability heuristic was analyzed.

**Results:**

The 6 HCI experts included professionals from the fields of nursing (n=4), pharmaceutical science (n=1), and systems engineering (n=1). The mean overall severity scores of the identified heuristic violations ranged from 0.66 (*flexibility and efficiency of use*) to 2.00 (*user control and freedom* and *error prevention*), in which scores closer to 0 indicate a more usable system. The heuristic principle *user control and freedom* was identified as the most in need of refinement and, particularly by nonnursing HCI experts, considered as having major usability problems. In response to the heuristic *match between system and the real world*, the experts pointed to the reversed direction of our system’s pain scale scores (1=severe pain) compared to those commonly used in clinical practice (typically 1=mild pain); although this was identified as a minor usability problem, its refinement was repeatedly emphasized by nursing HCI experts.

**Conclusions:**

Our heuristic evaluation process is simple and systematic and can be used at multiple stages of system development to reduce the time and cost needed to establish the usability of a system before its widespread implementation. Furthermore, heuristic evaluations can help organizations develop transparent reporting protocols for usability, as required by Title IV of the 21st Century Cures Act. Testing of EHRs and CDSSs by clinicians with HCI expertise in heuristic evaluation processes has the potential to reduce the frequency of testing while increasing its quality, which may reduce clinicians’ cognitive workload and errors and enhance the adoption of EHRs and CDSSs.

## Introduction

Despite the great potential of electronic health records (EHRs), clinicians are often confronted with unintended consequences related to the use of these systems, which can negatively impact patient safety [1-3]. One of the primary reasons for these unforeseen challenges stems from the lack of or poorly executed usability testing of these systems [4-6].

Usability measures the quality of a user’s experience when interacting with a system [7]. Recent evidence suggests that poor usability in EHRs is associated with an increase in clinicians’ cognitive workload, EHR-related fatigue, burnout, work inefficiency, job dissatisfaction, and intentions to leave the job [8-10]. System acceptance and adoption are crucial and strongly associated with the usability of EHR systems [11-13]. To optimize the benefits of EHRs for clinicians and avoid any unintended consequences that adversely impact patient safety, it is imperative to establish a system’s usability before its widespread implementation in real-world practice.

Usability evaluation methods are generally classified as expert- or user-based. Expert-based evaluations (eg, heuristic evaluations, cognitive walkthroughs, field observations) focus on ensuring that a system’s functionality is optimized and evidence-based interface standards and norms are met [14,15]. Evidence-based interface standards have been developed by various researchers to answer the following questions: (1) Does the user interface conform to evidence-based design principles? (2) Can users accomplish a given task? (3) Are users satisfied with the way a system helps perform a task? and (4) Can users operate the system efficiently with a quality outcome? [16-19]. In contrast, user-based evaluations focus on a user’s experience and interaction with a given system (eg, think-aloud method, interviews, focus groups, questionnaires) [14,15,20,21]. Although user-based usability testing shows differences in task performance between users who experienced difficulties and those who did not, expert-based usability testing focuses on “making things work” (ie, functionality) [12,14,20,22].

Clinical decision support systems (CDSSs) are specific components of EHRs that are frequently added and updated to reflect new evidence. CDSSs are defined as systems that provide clinicians with clinical knowledge and patient information that is “intelligently filtered and presented at appropriate times to improve patient care” [23]. When used as intended, CDSSs provide clinicians easy access to evidence-based information relevant to their decision-making process and can reduce their cognitive burden by minimizing the amount of information they must remember; these benefits enhance work efficiency, improve adherence to clinical guidelines, reduce the occurrence of medication errors, and prevent misdiagnoses [24-27]. Surprisingly, many CDSSs have not undergone rigorous usability and effectiveness testing prior to their deployment in practice [28]. The testing of CDSSs’ textual information and interfaces is critical in optimizing clinical decision-making and preventing errors in guidance.

A major challenge to establishing the usability of the CDSSs interfaced with EHRs has been the cost and time needed to carry out rigorous, iterative evaluations [21,29]. Attempting to fix usability issues after widespread deployment results in much higher costs than if done before implementation. Although usability studies should be iteratively conducted at multiple stages during system development [15], usability evaluations of health information technologies are often conducted during only a single stage of development [14]. In previous studies of CDSSs developed for clinicians that include nurses, usability testing was typically conducted either at an early stage for prototyping using an expert-based method [27,30] or after their deployment in practice using a user-based method [31-33]. Nurses participated in the evaluations mostly as a target user [31-33]; they may act as an expert—although they do not have usability expertise—after training by a usability expert to conduct the evaluation [27].

We believe that combining user- and expert-based evaluations has the potential to improve the efficiency and effectiveness of a system. In a user-based evaluation, the average cost per general user (ie, nonclinicians) is US $171, and at least twenty users are needed to identify 95% of the usability problems in a system [34,35]. Conducting iterative usability evaluations of EHRs and CDSSs with clinician-users is even more costly and time-consuming because recruiting them in clinical studies remains challenging [36,37]. In an expert-based evaluation, 3 to 5 expert evaluators are recommended [38], and 3 experts can identify 80%-90% of the usability problems [39]. Although both types of evaluation are valuable in testing EHRs and CDSSs [27,30-33], the stage of development often dictates the choice of the usability evaluation conducted. However, the predeployment phase, which occurs after prototyping, is the most crucial phase since eliminating usability issues in this phase avoids the costly fixes that will be needed if they are found after a system’s implementation [40,41]. Therefore, involving both experts and users in a late-stage (ie, predeployment stage after prototyping) usability evaluation would be optimal.

In this study, we offer an example application of our heuristic evaluation process, which provides a low-cost, time-effective, and expert-based method that includes potential users (ie, clinician usability experts) to evaluate the usability of CDSSs prior to their deployment in clinical practice.

## Methods

### Heuristic Evaluation

A heuristic evaluation is a usability-inspection method commonly used in the field of human-computer interaction (HCI) [16,21,38,39]. The heuristic evaluation proposed by Nielsen is an assessment conducted by a small group of evaluators using an evidence-based set of design guidelines called heuristics [38,42]. Heuristic evaluators, who are generally experts in HCI, examine a user interface and the system design according to the evidence-based interface standards.

### Example of Heuristic Evaluation Method

The example application of our approach involved the systematic evaluation of an electronic intervention containing clinical decision support (CDS) that was being prepared for deployment and testing by nurses in a national randomized controlled trial (RCT). Prior to nationwide deployment, we conducted a heuristic evaluation with HCI experts to identify any violations of usability principles in the CDS intervention.

We chose the heuristic evaluation process based on Nielsen’s 10 heuristics [42] and used a *heuristic evaluation checklist* developed by Bright et al [43]. The checklist facilitated each expert’s systematic inspection of the system’s user interface by judging its compliance with each usability factor through yes-or-no questioning and rating its overall severity for each of Nielsen’s 10 heuristics [42] on a scale of 0 (no problems) to 4 (usability catastrophe). Our heuristic evaluation process included specific HCI experts with nursing informatics expertise (referred to as “nursing HCI experts”) and general HCI experts (referred to as “nonnursing HCI experts”) to capture the views of both usability experts and clinician-users of our CDS intervention.

### CDS Intervention Under Evaluation

**Figure 1:**
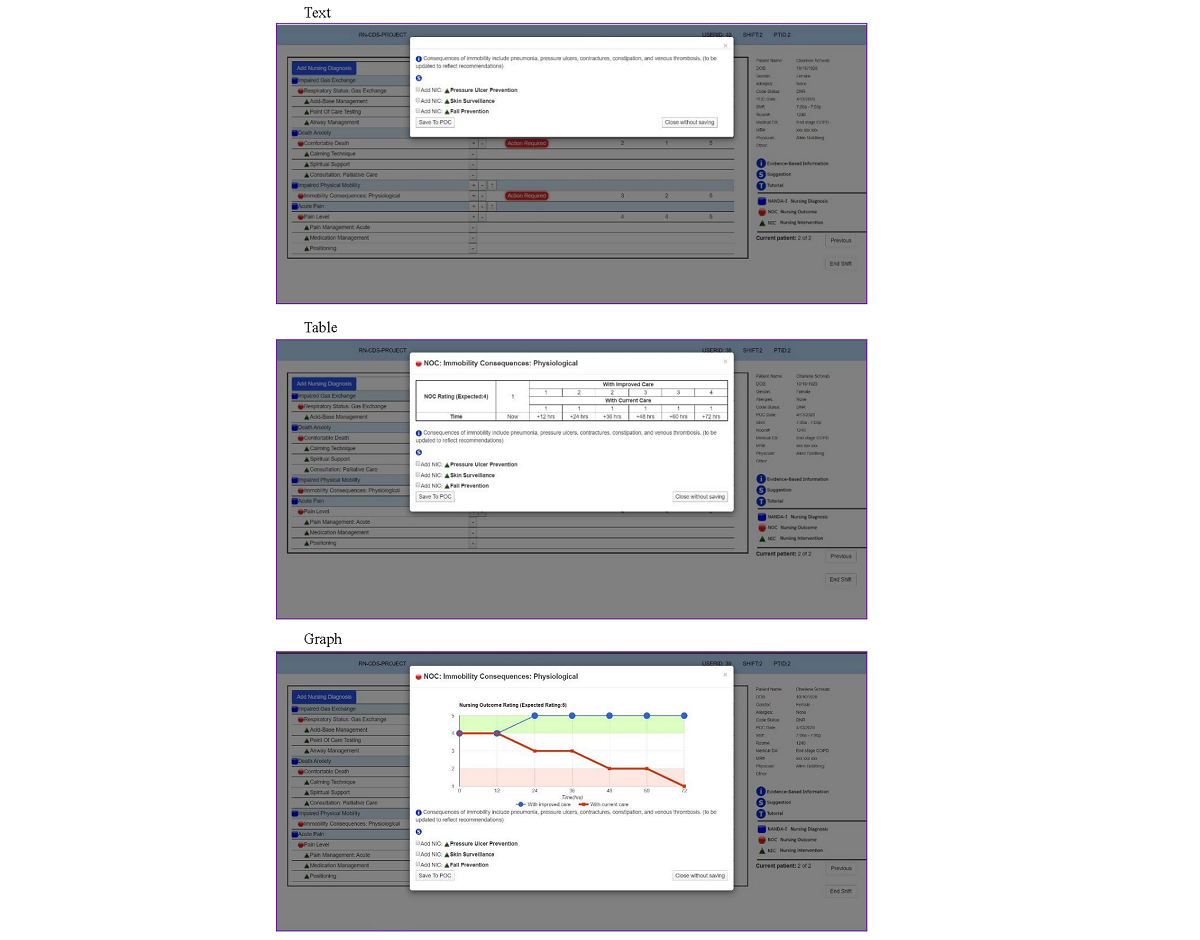
Three types of display formats (reproduced with permission from the HANDS Research Team). NANDA-I: NANDA International nursing diagnosis; NIC: nursing intervention classification; NOC: nursing outcome classification; POC: plan of care.

**Figure 2:**
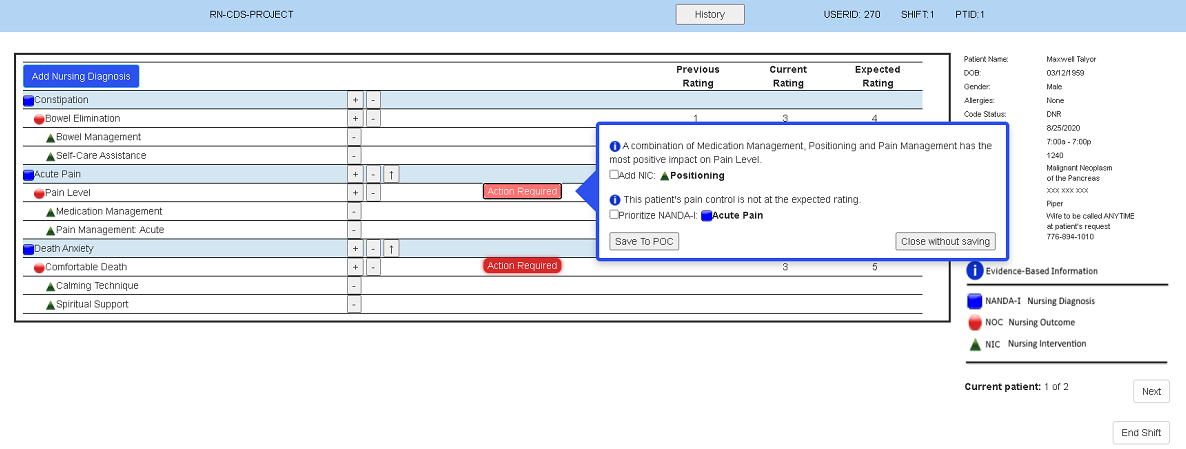
Clinical decision support suggestions (reproduced with permission from the HANDS Research Team). NANDA-I: NANDA International nursing diagnosis; NIC: nursing intervention classification; NOC: nursing outcome classification; POC: plan of care.

The main components of the CDS intervention evaluated in this paper were nursing diagnoses [44], nursing outcomes [45] with current and expected ratings, and nursing interventions [46]. Through an iterative design process with users (ie, nurses), our study team had previously developed and pretested a desktop prototype intervention designed to evaluate the effectiveness of 3 different electronic CDS intervention display formats: (1) text, (2) table, and (3) graph (see Figure 1) [47-50]. The CDS intervention contained evidence-based suggestions for improving palliative care delivered via a modular EHR care planning system (see Figure 2).

Subsequently, our team was funded by the National Institutes of Health to conduct a national, remotely administered RCT of the previously developed intervention. A desktop prototype in the 3 display formats (Figure 1) underwent iterative, user-centered–design usability studies with users (ie, user-based evaluations) [47-50]; however, a web-based application was needed to remotely test the CDS intervention with a national sample of 200 nurses. As small interface changes can impact the overall usability of an electronic CDS intervention, our team chose to conduct a second phase of usability testing using expert perspectives (ie, expert-based evaluations).

### Sampling and Recruitment

We used purposive sampling to invite 6 HCI experts, including nursing and nonnursing HCI experts, to participate in this study from August 3, 2020, to September 11, 2020. The sample size was decided in accordance with current recommendations, which state that including more than 3 to 5 evaluators in a heuristic evaluation is unlikely to yield additional useful information [38]. The main qualifications for participation were possession of a doctoral degree in the field of informatics and training in HCI. These qualifications were essential in this study since the quality of a heuristic evaluation is dependent on the skills and experience of the evaluators [22,51].

### Procedure

**Figure 3:**
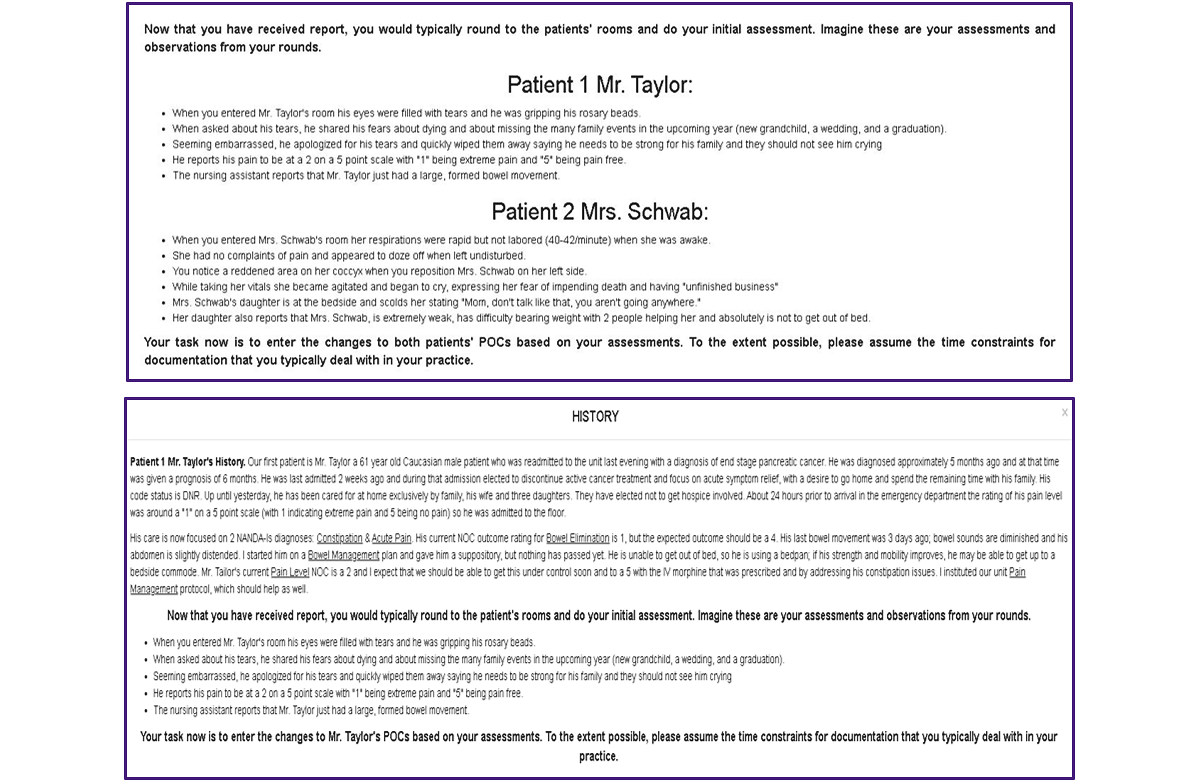
Use cases describing patient scenarios. POC: plan of care.

Our heuristic evaluation was conducted virtually during the COVID-19 pandemic. Before the evaluation, each expert was given a standardized orientation using a Microsoft PowerPoint video and transcript about how the CDS intervention works. The experts were also presented with the 2 use cases shown in Figure 3; these patient case scenarios require users (ie, nurses) to adjust their care plans to the unfolding clinical context. During the evaluation, each expert was asked to independently interact with the CDS intervention, ensuring unbiased evaluations from each evaluator. The experts were encouraged to explore the user interface of the entire CDS intervention at least twice.

After completing their given tasks using the use cases, each expert was asked to complete a *heuristic evaluation checklist* [42,43]. They were then asked to rate the overall severity of each identified heuristic violation on a scale of 0 to 4: 0 being no problems, 1 being a cosmetic problem only (ie, a fix can wait), 2 being a minor problem, 3 being a major problem, and 4 being a usability catastrophe (ie, requiring an immediate fix). Space was provided for the experts to add explanatory comments to identify the deficits of a usability factor and additional comments to justify each severity score. Since our upcoming clinical trial will test evidence-based suggestions using 3 information display formats (ie, text, table, and graph; see Figure 1), the *aesthetic and minimalist design* heuristic from the checklist was evaluated per display format.

### Ethics Approval

The University of Florida Institutional Review Board reviewed and approved the addition of an evaluation of the intervention software by experts, with no subjects in the clinical trial involved (IRB201902611).

### Data Analysis

Data analysis focused on the experts’ comments and overall severity scores collected via the *heuristic evaluation checklist*. To capture the experts’ perspectives on usability, we conducted deductive coding based on a pre-established set of guidelines (ie, heuristics). We developed a codebook for coding their comments using Microsoft Excel. Data from the coded comments were synthesized by 2 nursing informatics and HCI experts (HC and KDL), who were *not* participants in the heuristic evaluation, according to Nielsen’s 10 usability heuristics [38,42]. Differences in coding data were discussed until consensus was achieved.

Descriptive statistics were used to analyze the overall severity of the identified usability factors collected using the checklist. The mean and standard deviation of the overall severity score were calculated for each heuristic principle.

## Results

**Figure 4:**
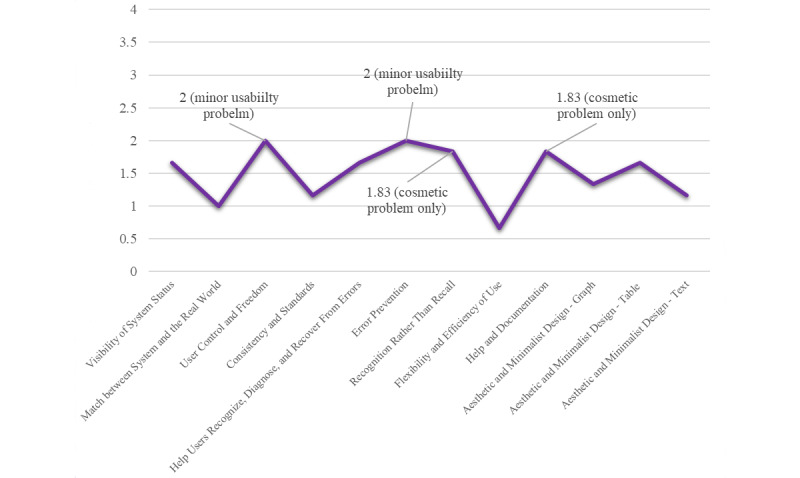
Four highest mean severity scores by heuristic. Severity score from 0 to 4: no usability problems (0), cosmetic problem only (1), minor usability problem (2), major usability problem (3), and usability catastrophe (4).

The 6 HCI experts who participated in the heuristic evaluation were professionals in the fields of nursing (n=4), pharmaceutical sciences (n=1), and system engineering (n=1). The mean overall severity scores of the identified heuristic violations ranged from 0.66 (*flexibility and efficiency of use*) to 2.00 (*user control and freedom* and *error prevention*), in which scores closer to 0 indicate a more usable system. Figure 4 depicts the mean severity scores by heuristics and highlights the 4 highest scores. Table 1 organizes the evaluation’s mean severity scores and sample comments into Nielsen’s 10 usability heuristics.

The heuristic principles identified as the most in need of refinement were *user control and freedom* (mean 2.00, SD 1.09) and *error prevention* (mean 2.00, SD 1.09). Although all heuristics were identified as having major (ie, severity score of 3) and minor (ie, severity score of 2) usability problems, *user control and freedom* was considered a major usability issue particularly by nonnursing HCI experts, who pointed out that users of the CDS intervention were unable to alter current and expected scores for nursing outcomes once the ratings were entered in. To improve this heuristic, the experts suggested that the “Undo” function should not be limited and to give users the ability to fix the entered scores. Similarly, after the “Action Required” menu was completed, it was no longer possible for users to select the “Undo” function to bring it up again. An example of this is shown in Figure 2, where the “Action Required” was choosing nursing interventions for the plan of care (POC) based on the decision support suggestions recommended by our CDS intervention.

**Table 1:** Mean severity scores and sample comments from the heuristic evaluations.

Usability heuristic		Severity score^a^, mean (SD)	Sample comments
Visibility of system status		1.66 (1.21)	Unclear if care plan icons (circle, square, triangle) are clickable
Match between system and the real world		1.00 (1.09)	Pain scale in the CDS intervention, in which score 1 indicates “severe” pain is the opposite of common pain scales used in clinical practice (1 indicates “mild” pain)
User control and freedom		2.00 (1.09)	Limited “Undo” functionality
Consistency and standards		1.16 (1.16)	Unclear of formatting standards referred
Help users recognize, diagnose, and recover from errors		1.66 (1.63)	Error message is not informative as it doesn’t indicate where the error occurred
Error prevention		2.00 (1.09)	Need a warning message when clicking the minus button
Recognition rather than recall		1.83 (1.16)	Unclear what was undone
Flexibility and efficiency of use		0.66 (1.03)	Suggested helping users to find content on the site (hyperlinks, alphabetical index)
Help and documentation		1.83 (0.98)	Needs HELP function to inform on how the CDS intervention works
**Aesthetic and minimalist design**			
	Graph format	1.33 (1.50)	Not visually appealing from similar blues/grey shades
	Table format	1.66 (1.50)	Font is too small and difficult to read
	Text format	1.16 (0.75)	No labels in the icons Suggested we use text section headers instead of icons

^a^Severity score from 0=best to 4=worst: no usability problems (0), cosmetic problem only (1), minor usability problem (2), major usability problem (3), and usability catastrophe (4).

**Figure 5:**
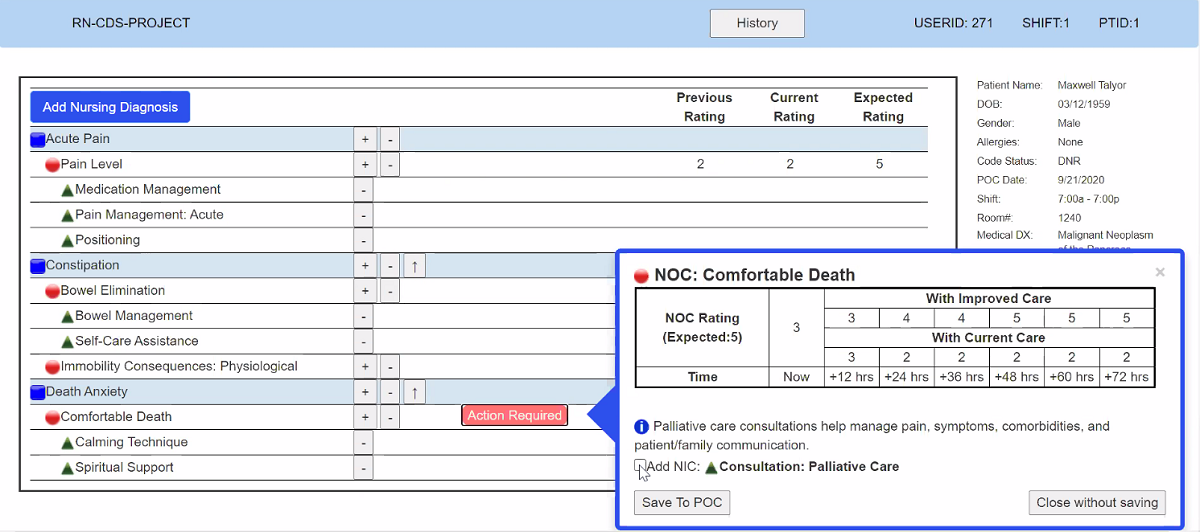
Action Required menu (reproduced with permission from the HANDS Research Team). NIC: nursing intervention classification; NOC: nursing outcome classification; POC: plan of care.

**Figure 6:**
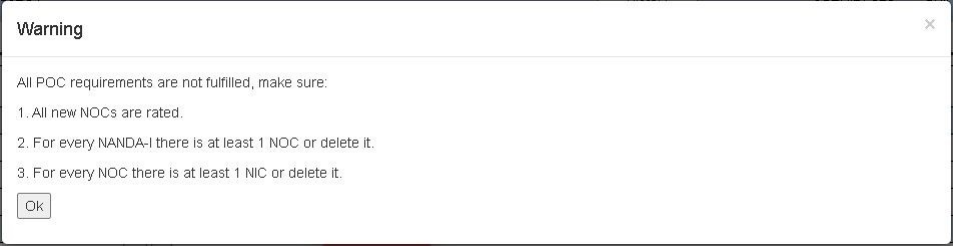
Warning message (reproduced with permission from the HANDS Research Team). NANDA-I: NANDA International nursing diagnosis; NIC: nursing intervention classification; NOC: nursing outcome classification; POC: plan of care.

In response to *error prevention*, the experts found the exit (x) button in the upper right corner of the “Action Required” menu to be confusing since 2 other options are also available: “Save To POC” and “Close without saving” in the lower left and right corners of the screen, respectively (Figure 5). To improve *error prevention*, the experts suggested that we provide the warning message shown in Figure 6 when the minus button is clicked; they also recommended that this warning message indicate where the error occurred to support the heuristic *help users recognize, diagnose, and recover from errors* (mean 1.66, SD 1.63).

The next heuristics identified as requiring the most improvement were *recognition rather than recall* (mean 1.83, SD 1.16) and *help and documentation* (mean 1.83, SD 0.98). *Recognition rather than recall* was considered a major usability problem particularly by nonnursing HCI experts, who stated that clicking the “Undo” button to see what was undone should be recognizable to users. Regarding *help and documentation*, the experts emphasized the need for a “Help” or “Search” functionality to inform users of how our CDS intervention works (eg, how users can add a new nursing diagnosis) and reduce user errors when using the intervention.

**Figure 7:**
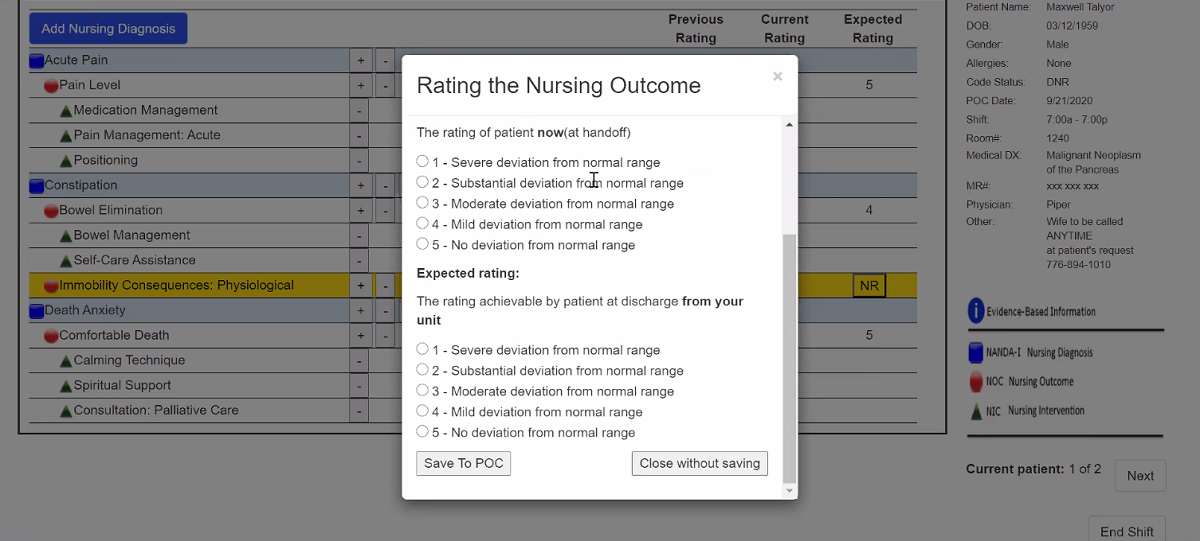
Pain scale scores (reproduced with permission from the HANDS Research Team). NANDA-I: NANDA International nursing diagnosis; NIC: nursing intervention classification; NOC: nursing outcome classification; POC: plan of care.

Finally, for the heuristic *match between system and the real world* (mean 1.00, SD 1.09), the experts pointed to the reversed direction of our pain scale scores (1 indicating severe pain) compared to those commonly used in clinical practice (1 indicating mild pain; Figure 7). Although this usability issue was identified as minor, its refinement was repeatedly emphasized by nursing HCI experts.

## Discussion

### Principal Findings

With the proliferation of EHRs and CDSSs in today’s health care, rigorous and multistage usability evaluations are essential to the development of effective electronic systems; however, these evaluations are considered challenging due to the cost and time required to conduct them [21,29]. In this study, we provided an example application of a heuristic evaluation process that we used prior to the deployment of an electronic CDS intervention for our RCT study. The same process can be used with different EHRs and CDSSs and at multiple phases of development to provide high-quality, low-cost, and efficient usability assessments. This heuristic evaluation method can also help organizations develop transparent reporting on a system’s usability, as required by Title IV of the 21st Century Cures Act [52]. As evidenced in this study, conducting this evaluation enabled us to detect unmet evidence-based usability principles of an electronic CDS intervention prior to its deployment.

This study took approximately 2 months (from August to September 2020) to locate and enlist the experts, distribute study materials, and compile the results. It is important to emphasize that this study was conducted during the global COVID-19 pandemic, which potentially affected the recruitment period as well as data collection. Thus, our process can likely be performed in a shorter period of time than the 2 months we experienced.

Through expert-based usability testing, we discovered major and minor usability problems in the user interface of an electronic CDS intervention prior to its deployment for use by users. Despite their benefits, heuristic evaluations are rarely reported for usability testing, especially in late-stage (ie, predeployment stage after prototyping) usability testing. Although user-based usability testing is effective in identifying major usability issues that affect user performance, a focus on user testing alone may lead to missed usability violations that users who do not have HCI expertise may not recognize [53-55]. Although unrecognized, these violations can decrease the system’s usability, increase users’ cognitive workload, create unintended consequences that threaten patient safety, and result in the EHR and CDSS being discontinued in practice. Future work should include a reevaluation of the CDS intervention after the recommendations against the heuristic violations have been implemented. In summary, heuristic evaluations have the potential to clarify usability issues within EHRs and CDSSs, not only after deployment but also before deployment, since they can be employed throughout various stages of system development [56]. Thus, this study reveals the value of including expert review methods at some point during the development process to ultimately achieve the goals of the system.

A heuristic evaluation with experts can identify minor usability problems that are often not detected in user testing but can be costly to fix if detected after a system’s deployment [39]. Fixing usability problems after deployment or during maintenance stages usually costs 40 to 100 times more than fixing them before deployment and in the development stage [40,41]; therefore, the early refinement of CDSSs using a heuristic evaluation process, such as the one described in this paper, ultimately reduces a system’s overall development and redesign costs.

Since expert-based usability testing focuses on “making things work” in a natural and logical order, the experts in this study recommended changing the direction of our intervention’s pain scale to range from 0 (no pain) to 4 (severe); this pain scale now matches those used in real-world clinical practice and would be intuitive to use. It is important to note that this usability problem was detected only by nursing HCI experts who have backgrounds in clinical nursing practice; this underscores the advantage of having a panel of experts who boasts skills and experience in the relevant clinical domains (eg, nursing, medicine), as well as in usability and HCI, when evaluating clinical technologies [51]. Our purposively selected panel of HCI experts, including nursing and nonnursing HCI experts, enabled us to identify significant usability problems that may have increased the likelihood of medical errors in real-world clinical settings, which is an important strength of this study.

### Limitations

The limitations of this study were related to the experts’ independent evaluations. To complete the evaluation, each expert used his or her own device (eg, desktop and laptop computers, tablets) with differing screen sizes; this could have influenced their evaluations of the CDS intervention. Nonetheless, to obtain an optimal idea of the intervention’s general scope, we asked the experts to use Google Chrome’s Incognito (ie, private) browser to access the intervention, as well as to carefully explore the user interface’s screen layout and interaction structure at least twice [20].

Another potential limitation of our study is that we did not collect the demographic information of our study participants. We invited them to participate in our expert-based evaluation as HCI experts either with or without domain expertise.

### Conclusions

Our heuristic evaluation process is simple, systematic, and theoretical and can ensure a system’s optimal functionality. Beyond confirming that evidence-based interface standards and norms are met, our process can be used at multiple stages of system development before implementation (ie, predeployment phase after prototyping) to reduce the time and cost of the iterative evaluations needed to improve a system’s usability after widespread implementation. A heuristic evaluation that includes HCI experts with domain expertise (ie, clinician HCI experts) has the potential to reduce the frequency of testing while increasing its quality, which may reduce clinicians’ cognitive workload and EHR-related errors. Making this small investment in early refinement can reap sizable benefits to further enhance EHR and CDSS adoption and acceptance by various clinicians in real-world clinical practice.

## Abbreviations

CDS: clinical decision support
CDSS: clinical decision support system
EHR: electronic health record
HCI: human-computer interaction
POC: plan of care
RCT: randomized controlled trial
